# Complete chloroplast genome of a rare and endangered plant species *Phalaenopsis zhejiangensis*: genomic features and phylogenetic relationship within Orchidaceae

**DOI:** 10.1080/23802359.2021.1972049

**Published:** 2021-09-09

**Authors:** Ming Jiang, Yan Zhu, Qian Wu, Huijuan Zhang

**Affiliations:** College of Life Sciences, Taizhou University, Taizhou, PR China

**Keywords:** *Phalaenopsis zhejiangensis*, rare and endangered species, chloroplast genome, phylogenetic analysis

## Abstract

*Phalaenopsis zhejiangensis* is a rare and endangered plant species with extremely small populations. The complete chloroplast (cp) genome of *P. zhejiangensis* was assembled, its structural organization was described and comparative genomic analyses was carried out. The cp genome of *P. zhejiangensis* is 143,547 bp in length, with a GC content of 37.2%, which includes a pair of inverted repeats (IRs) of 24,464 bp separated by a small single-copy region of 10,764 bp and a large single-copy region of 83,856 bp. The cp genome contains 126 genes, consisting of 80 protein-coding genes, 38 transfer RNAs, and eight ribosomal RNAs. Six protein-coding genes, including *ψndhB* (two copies), *ψndhD*, *ψndhG*, *ψndhK*, and *ψndhI*, are identified as pseudogenes. Another six *ndh* genes, *ndhA*, *ndhC*, *ndhE*, *ndhF*, *ndhH*, and *ndhJ*, are missing from the plastid genome. A total of 41 cp simple sequence repeats (SSRs) were identified, including 40 mono-nucleotides and one di-nucleotides. Phylogenic analysis revealed *P. zhejiangensis* was nested inside the *Phalaenopsis* species and sister to *P. wilsonii*. The assembly and analysis of *P. zhejiangensis* cp genome will provide essential data for further study of taxonomy and systematics of Orchidaceae.

## Introduction

Orchidaceae, a family of monocotyledonous plants, is one of the most successful and highly evolved monocots, comprising 763 genera with approximately 28,000 species (Hadi et al. 2015; Christenhusz and Byng 2016). The genus *Phalaenopsis* belongs to tribe Vandeae, which is commonly known as moth orchids, including more than 70 species widely distributed across Southeast Asia to northern Australia, with the majority in Indonesia and the Philippines (Ko et al. 2017; Gogoi and Rinya 2020; Lee et al. 2020). There are 12 *Phalaenopsis* species distributed in China, among which four species are endemics (Wu et al. 2009). *Phalaenopsis zhejiangensis* is a small epiphytic plant with slightly flattened roots, very short stems, basal leaves, and thinly textured flowers (Wu et al. 2009). *P. zhejiangensis* was transferred from *Doritis zhejiangensis*, while the latter was renamed from *Nothodoritis zhejiangensis* (Yukawa and Kita 2005), which was first found on a trunk of *Podocarpus macrophyllus* in 1970 at Xitianmu Mountain in Linan County, Zhejiang Province (Tsi 1989; Schuiteman 2012). *P. zhejiangensis* is a rare and endangered plant species in China, which is narrowly distributed in provinces of Zhejiang Province, Gansu, Anhui, and Shanxi, growing on the bark of trees or rock surfaces, with extremely small populations. As a result of habitat loss, *P. zhejiangensis* is listed as threatened with extinction. Fortunately, Zeng et al (2011) have established an effective propagation protocol for large-scale propagation of this endangered orchid species. In recent years, several chloroplast (cp) genomes of *Phalaenopsis* plants, including *P. aphrodite*, *Phalaenopsis* ‘Tiny Star’, *P. lowii*, *P*. *lobbii*, and *P. mannii*, were sequenced and assembled (Chang et al. 2006; Kim et al. 2016; Wang et al. 2019; Chen et al. 2020; Zhang et al. 2020). However, the cp genome of *P. zhejiangensis* has not yet been assembled. In our study, the complete cp genome sequence of *P. zhejiangensis* was assembled to provide new insights into taxonomy and systematics of Orchidaceae.

## Materials and methods

### Plant material and DNA extraction

Fresh leaves were collected from a plant nursery (28°65.762′N, 121°46.976′E) in Jiaojiang, Zhejiang Province, China. A voucher specimen was deposited at the Molecular Biology Laboratory in Taizhou University (http://www.tzc.edu.cn, Dr. Huijuan Zhang, zhanghj82@126.com) under a collection number of CHS2020029. Fresh leaves were ground into a fine powder in liquid nitrogen with a mortar and pestle, and genomic DNA was extracted following the CTAB-based protocol as described by Doyle and Doyle (1987).

### DNA sequencing and sequence assembly

Construction of a genomic DNA library was carried out according to the manufacturer’s instructions, and high-throughput sequencing was then performed using the Illumina Hiseq X Ten system (Illumina, San Diego, CA). Approximately, 3.64 Gb raw data of 150 bp paired-end Illumina reads were generated, and 3.63 Gb high-quality clean reads were harvested by using the NGS QC Toolkit v2.3.3 (Patel and Jain 2012). Geneious Prime 2019 (Biomatters, Auckland, New Zealand) was employed to assemble the complete cp genome.

### Chloroplast genome annotation

Annotation of the complete cp genome was performed with Geneious Prime 2019 by using *P. lobbii* as a reference genome (NCBI accession number: MT830847) (Wang et al. 2019), and the borders of each gene were manually inspected and corrected where necessary. The circular cp genome map of *P. zhejiangensis* was drawn using the online program OrganellarGenomeDRAW (OGDRAW, http://ogdraw.mpimp-golm.mpg.de) with default settings (Lohse et al. 2013).

### Simple sequence repeat analysis

Perl scripts of MIcroSAtellite Identification Tool (MISA) were used to identify and locate the potential simple sequence repeats (SSRs) loci in complete cp genome sequence of *P. zhejiangensis* (Thiel et al. 2003). To determine the presence of SSRs, 1–6 bp nucleotide motifs were considered, and the minimum repeat numbers were set as 10 repeat units for mono-nucleotides, six for di-nucleotides, and five for tri-, tetra-, penta-, and hexa-nucleotides.

### Phylogenetic analysis

The complete cp genome sequences of 39 Orchidaceae species downloaded from NCBI were used for phylogenetic analysis, which included eight *Phalaenopsis* plants, six *Holcoglossum* species, and five *Cleisomeria* plants. The complete cp genome of *Coix lacryma-jobi* (FJ261955) was applied as an outgroup. A total of 41 genome sequences were aligned with a multiple sequence alignment program MAFFT v7.388 plugin in Geneious Prime 2019 under default settings (Katoh and Standley 2013). The best-fit evolutionary model of DNA substitution for maximum-likelihood (ML) analysis was determined by using jModelTest 2.1.9 under the Akaike information criterion (AIC) (Darriba et al. 2012). A phylogenetic tree was generated by PhyML 3.1, with 1000 bootstrap replicates (Guindon et al. 2010).

## Results

### Genome sequencing and assembly

**Figure 1.**
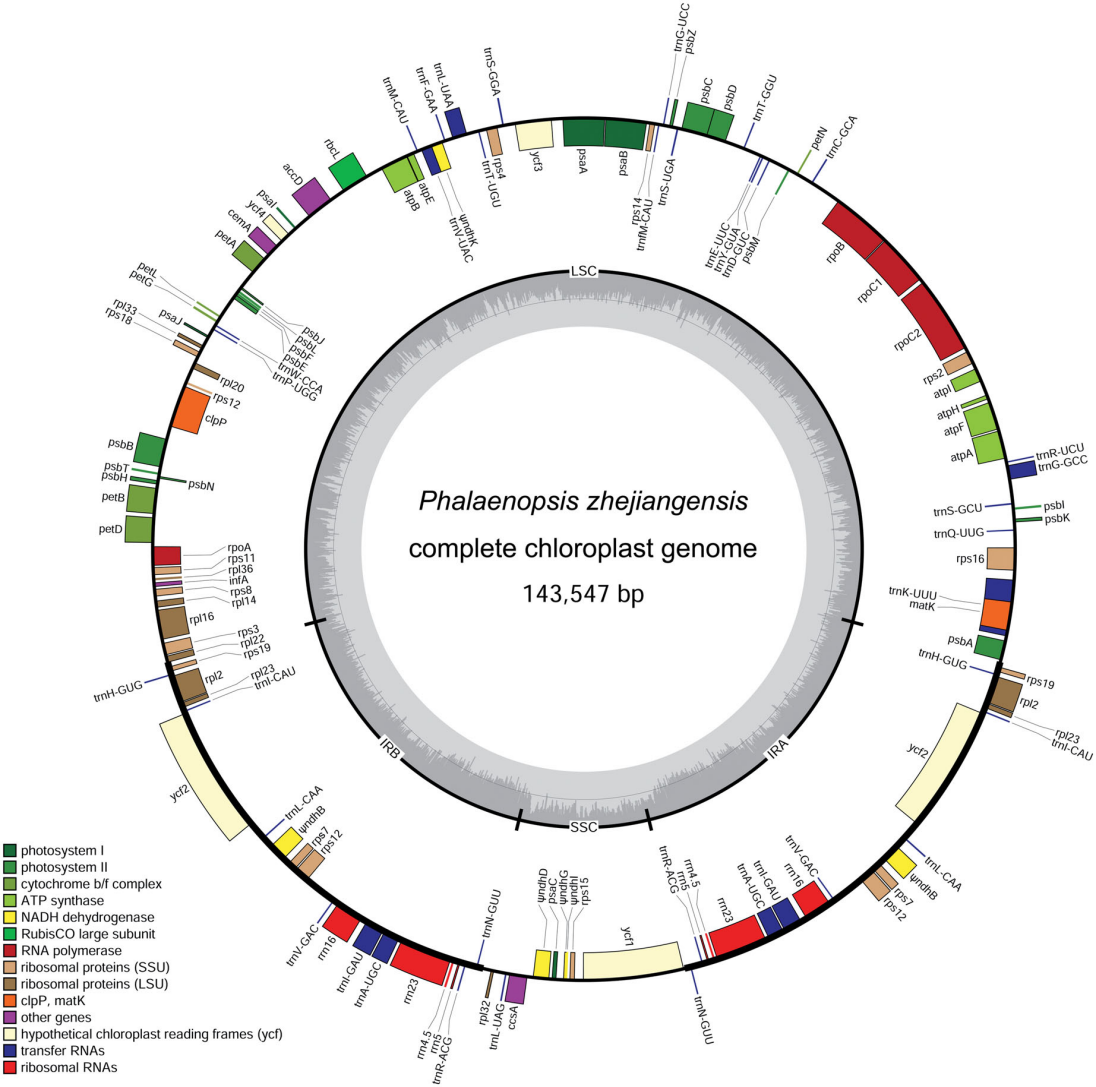
The chloroplast genome of *Phalaenopsis zhejiangensis*. From the center going outward, the four circles indicate scattered forward and reverse repeats, tandem repeats, microsatellite sequences identified, and gene structure of the plastome.

More than 12 million paired-end clean reads were obtained, and a circular cp genome was assembled with Geneious Prime. The complete cp plastome of *P. zhejiangensis* is 143,547 bp in length which includes a pair of inverted repeats (IRs) of 24,464 bp separated by a large single-copy (LSC) region of 83,855 bp and a small single-copy (SSC) region of 10,764 bp (Figure 1). The overall guanine–cytosine (GC) content of *P. zhejiangensis* cp genome is 36.8%, while the corresponding values of LSC, SSC, and IR sequences are 34.0%, 28%, and 43.5%, respectively.

### Genome features of *Phalaenopsis zhejiangensis*

The complete cp plastome of *P. zhejiangensis* is comprised of 126 genes, including 74 protein-coding genes, 38 tRNA genes, eight rRNA genes, and six pseudogenes. All the six pseudogenes are *ndh*s, including *ψndhD*, *ψndhG*, *ψndhI*, *ψndhK*, and two copies of *ψndhB*. Additionally, other six *ndh* genes have been lost in the plastome, these are *ndhA*, *ndhC*, *ndhE*, *ndhF*, *ndhH*, and *ndhJ*. Six tRNA genes (*trnA-UGC*, *trnH-GUG*, *trnI-CAU*, *trnI-GAU*, *trnL-CAA*, *trnN-GUU*, *trnR-ACG*, and *trnV-GAC*) and four rRNA genes (*rrn4.5*, *rrn5*, *rrn16*, and *rrn23*) are present in two copies. Among the protein-coding genes, eight genes including *rpl16*, *rps16*, *rpoC1*, *petB*, *petD*, *atpF*, and two copies of *rpl2* contain a single intron, while three genes, *rps12*, *ycf3*, and *clpP*, contain two introns. Moreover, six tRNA genes, *trnA-UGC*, *trnG-GCC*, *trnI-GAU*, *trnK-UUU*, *trnL-UAA*, and *trnV-UAC*, harbored a single intron have been identified (Table 1).

**Table 1.** List of genes in the chloroplast genome of *Phalaenopsis zhejiangensis*.

Group of genes	Name of genes	Total number
Large subunit of ribosomal proteins	*rpl2* (×2)*, *rpl14*, rpl16*, *rpl20*, *rpl22*, *rpl23* (×2), *rpl32*, *rpl33*, *rpl36*	11
Small subunit of ribosomal proteins	*rps2*, *rps3*, *rps4*, *rps7* (×2), *rps8*, *rps11*, *rps12* (×2)**, *rps14*, *rps15*, rps16*, *rps18*, *rps19*(×2)	15
DNA-dependent RNA polymerase	*rpoA*, *rpoB*, rpoC1*, *rpoC2*	4
Ribosomal RNA genes	*rrn4.5* (×2), *rrn5* (×2), *rrn16* (×2), *rrn23* (×2)	8
Transfer RNA genes	*trnA-UGC* (×2)*, *trnC-GCA*, *trnD-GUC*, *trnE-UUC*, *trnF-GAA*, *trnfM-CAU*, trnG-GCC*, *trnG-GCC, trnH-GUG* (×2), *trnI-CAU* (×2), *trnI-GAU* (×2)*, trnK-UUU*, *trnL-CAA* (×2), trnL-UAA*, *trnL-UAG*, *trnM-CAU*, *trnN-GUU* (×2), *trnP-UGG*, *trnQ-UUG*, *trnR-ACG* (×2), *trnR-UCU*, *trnS-GCU*, *trnS-GGA*, *trnS-UGA*, *trnT-GGU*, *trnT-UGU*, *trnV-GAC* (×2), trnV-UAC*, *trnW-CCA*, *trnY-GUA*	38
Photosystem I	*psaA*, *psaB*, *psaC*, *psaI*, psaJ, ycf3**	6
Photosystem II	*psbA*, *psbB*, *psbC*, *psbD*, *psbE*, *psbF*, *psbH*, *psbI*, *psbJ*, *psbK*, *psbL*, *psbM*, *psbN*, *psbT*, *psbZ*	15
Cytochrome b6/f complex	*petA*, petB*, petD*, *petG*, *petL*, *petN*	6
NADH dehydrogenase	ψ*ndhB (×2)*, ψ*ndhD*, ψ*ndhG*, ψ*ndhI*, ψ*ndhK*	6
ATP synthase	*atpA*, *atpB*, *atpE*, atpF*, *atpH*, *atpI*	6
Rubisco	*rbcL*	1
Translational initiation factor	*infA*	1
Maturase	*matK*	1
Protease subunit P	clpP**	1
Envelop membrane protein	*cemA*	1
Subunit acetyl-CoA carboxylate	*accD*	1
c-type cytochrome synthesis gene	*ccsA*	1
Conserved open reading frames	*ycf1*, *ycf2 (×2)*, *ycf4*	4
Total		126

(ψ) pseudogene; *one intron; **two introns.

### IR expansion and contraction

**Figure 2.**
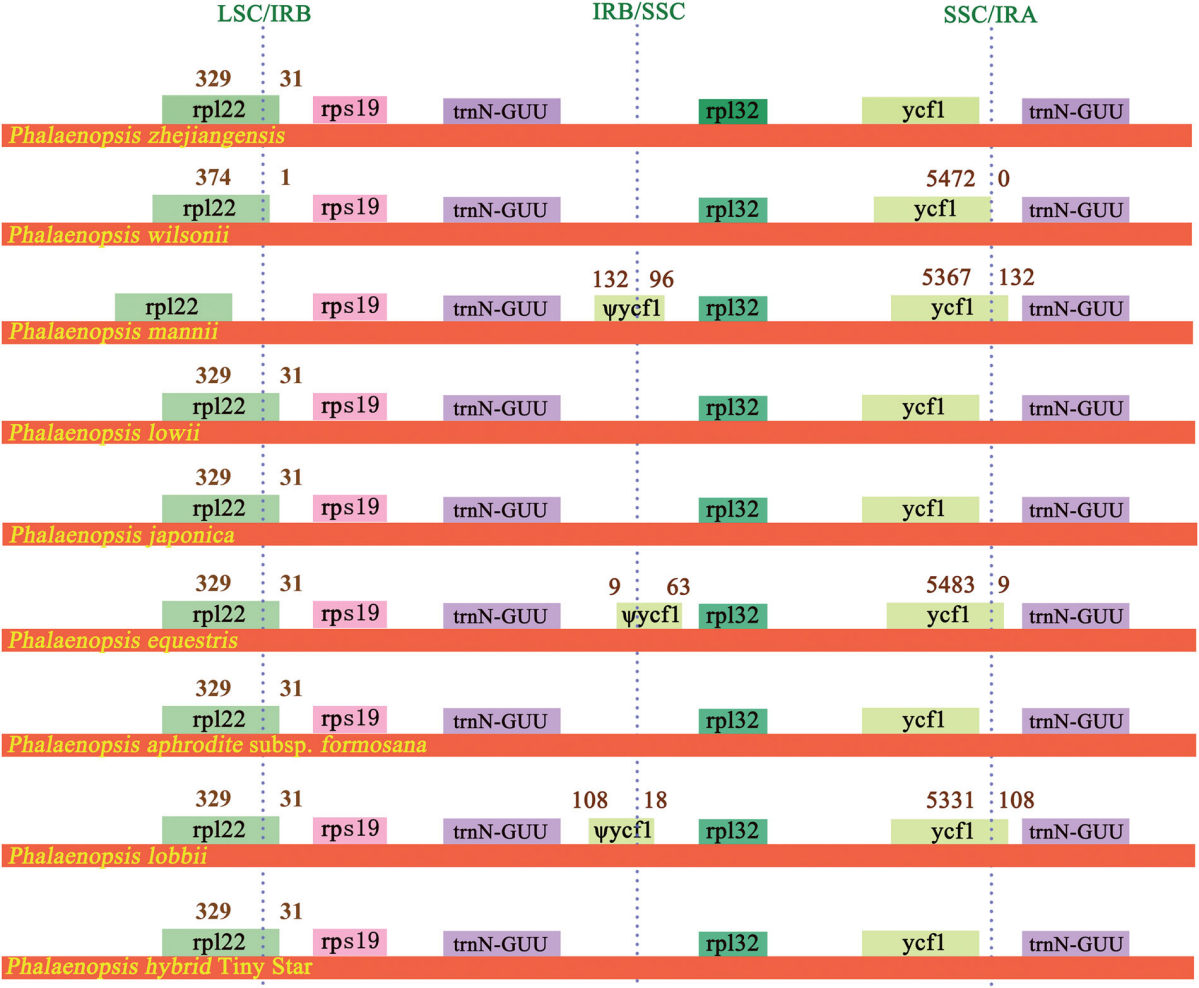
Structural variations at inverted-repeat and single-copy borders in nine *Phalaenopsis* chloroplast genomes. The figure features are not to scale regarding sequence length.

The IR region of *P. zhejiangensis* is 24,464 bp in length, while the other eight *Phalaenopsis* IRs included in our study are longer than *P. zhejiangensis*, they range from 24,719 bp to 25,846 bp (Table 2). Comparisons of LSC, IRB, SSC, and IRA junction boundaries among nine *Phalaenopsis* cp genomes were performed and presented in Figure 2. In *Phalaenopsis* plastome, LSC/IRB boundary junctions lie within *rpl22* gene except *P. mannii*, which locates in the intergenic region between *rpl22* and *rps19*. Eight *rpl12* genes extend 31 bp into IRB regions, while the *rpl12* in *P. wilsonii* extends only one base pair into its IRB region (Figure 2). The IRB/SSC junctions of *P. mannii*, *P. equestris*, and *P. lobbi* locate within the *ψycf1* pseudogenes; however, no *ψycf1* is identified in other six *Phalaenopsis* cp genomes including *P. zhejiangensis*. The SSC/IRA junctions of *P. wilsonii*, *P. mannii*, *P. equestris*, and *P. lobbii* lie within *ycf1* genes, which extend 0 bp, 132 bp, 9 bp, and 108 bp into IRA regions, respectively while the SSC/IRA junctions of *P. zhejiangensis*, *P. lowii*, *P. japonica*, *P. aphrodite* subsp. *formosana*, and the hybrid ‘Tiny Star’ locate between *ycf1* and *trnN-GUU*.

**Table 2.** Details of nine *Phalaenopsis* chloroplast genomes.

Plant species	Accession number in NCBI	Genome length (bp)	GC contents (%)	LSC (bp)	SSC (bp)	IR (bp)
*Phalaenopsis aphrodite* subsp. *formosana*	AY916449	148,964	36.7	85,957	11,543	25,732
*Phalaenopsis equestris*	JF719062	148,959	36.7	85,967	11,300	25,846
*Phalaenopsis hybrid* ‘Tiny Star’	NC_025593	148,918	36.7	85,885	11,523	25,755
*Phalaenopsis japonica*	NC_046808	146,942	36.8	84,882	10,568	25,746
*Phalaenopsis lobbii*	MT830847	144,607	36.6	83,482	11,687	24,719
*Phalaenopsis lowii*	NC_050652	146,834	36.9	84,616	10,473	25,633
*Phalaenopsis mannii*	NC_050940	148,596	36.7	85,300	11,640	25,828
*Phalaenopsis wilsonii*	MW218959	145,373	36.9	84,995	10,668	24,855
*Phalaenopsis zhejiangensis*	MZ326749	143,574	38.7	83,854	10,764	24,464

### SSR analysis

Totally, *P. zhejiangensis* cp genome contains 41 SSRs (Table 3). Among the SSRs identified herein, A/T mononucleotide repeats account for the largest proportion of 97.56%. Only one di-nucleotide repeat was observed, and no tri-, tetra-, penta-, or hexa-nucleotides presented in the plastome. Most SSRs are distributed in LSC (70.73%), followed by SSC (24.39%), and the proportion in IRs is less than 5%. Among 41 SSRs, 25 loci lie in intergenic regions, four in introns, and 12 in coding regions or pseudogenes.

**Table 3.** Repeat sequences and their distribution within *Phalaenopsis zhejiangensis* chloroplast genome.

Number	Motif	Size	Start	End	Location
1	(T)10	10	4245	4254	IGS
2	(A)10	10	7693	7702	IGS
3	(T)10	10	9184	9193	IGS
4	(T)10	10	9599	9608	IGS
5	(T)17	17	14,113	14,129	IGS
6	(T)12	12	17,053	17,064	IGS
7	(A)16	16	27,913	27,928	IGS
8	(T)10	10	28,756	28,765	IGS
9	(A)11	11	32,765	32,775	IGS
10	(T)10	10	33,072	33,081	IGS
11	(A)10	10	33,357	33,366	IGS
12	(A)10	10	37,057	37,066	IGS
13	(A)11	11	47,194	47,204	IGS
14	(A)10	10	47,875	47,884	IGS
15	(A)11	11	56,140	56,150	IGS
16	(T)12	12	62,492	62,503	IGS
17	(A)10	10	64,380	64,389	IGS
18	(T)10	10	64,612	64,621	IGS
19	(T)10	10	71,393	71,402	IGS
20	(T)10	10	79,492	79,501	IGS
21	(T)10	10	83,505	83,514	IGS
22	(T)10	10	98,214	98,223	IGS
23	(A)11	11	108,703	108,713	IGS
24	(T)10	10	113,355	113,364	IGS
25	(A)10	10	129,180	129,189	IGS
26	(A)10	10	111,423	111,432	*ψndhD*
27	(A)10	10	111,579	111,588	*ψndhD*
28	(TA)8	16	111,722	111,737	*ψndhD*
29	(T)10	10	49,573	49,582	*ψndhK*
30	(A)12	12	74,999	75,010	*petB* intron
31	(T)10	10	82,386	82,395	*rpl16* intron
32	(A)11	11	23,345	23,355	*rpoC1* intron
33	(T)11	11	69,574	69,584	*clpP*
34	(A)10	10	69,867	69,876	*rps12*
35	(T)10	10	9805	9814	*trnG-GCC* intron
36	(T)12	12	114,415	114,426	*ycf1*
37	(T)11	11	115,271	115,281	*ycf1*
38	(T)13	13	116,348	116,360	*ycf1*
39	(T)10	10	117,578	117,587	*ycf1*
40	(T)10	10	117,695	117,704	*ycf1*
41	(A)13	13	45,453	45,465	*ycf3*

IGS: intergenic spacer region.

### Phylogenetic analysis

**Figure 3.**
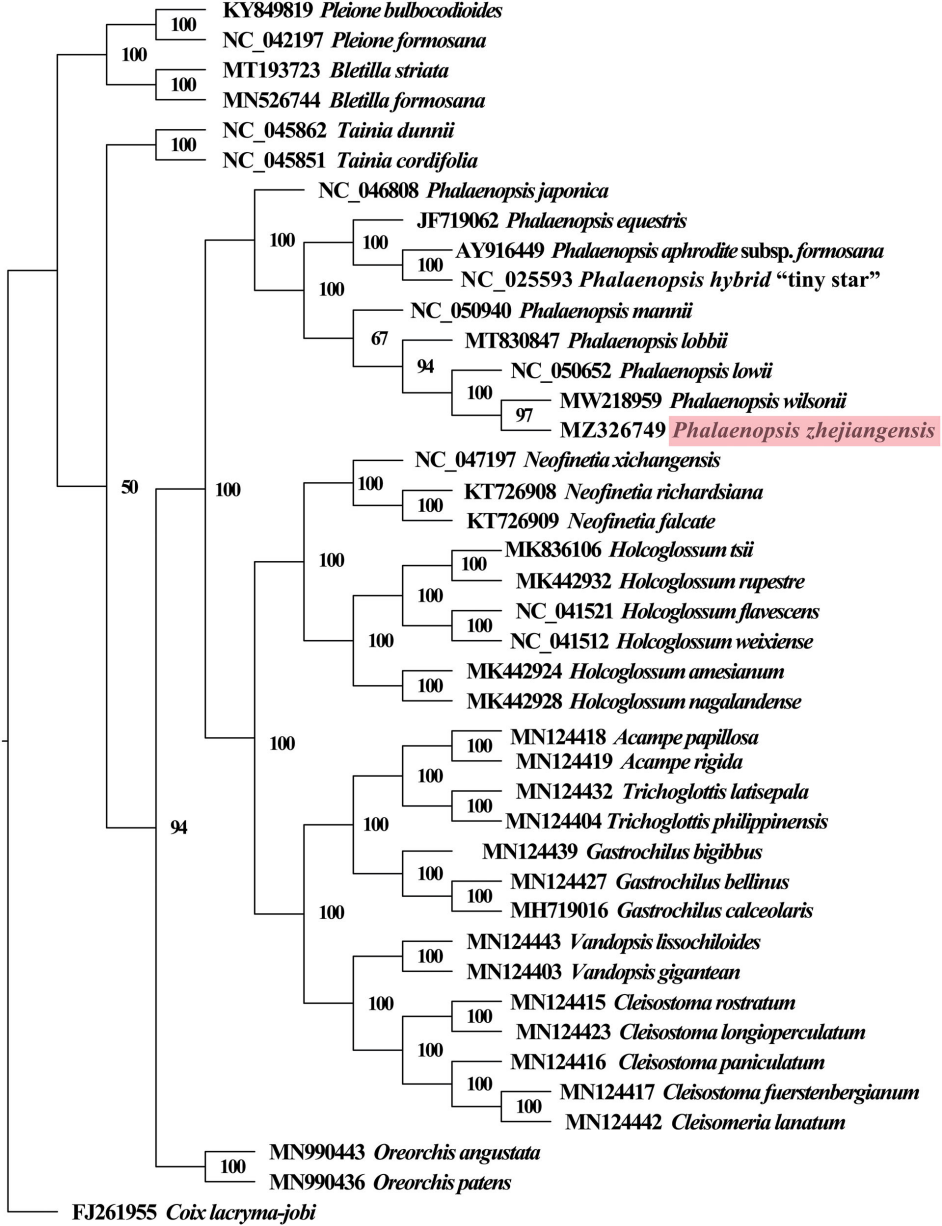
The maximum-likelihood tree inferred from 41 Orchidaceae complete chloroplast genomes under GTR + G+I model with maximum-likelihood value (Llk)=–604333.07383, Akaike information criterion (AIC)=1208844.14766, and Bayesian information criterion (BIC)=1209758.57750.

Statistical selection of the best-fit model of nucleotide substitution was carried out by the jModelTest program, and GTR + G+I was turned out to be the best-fitted. To evaluate the phylogenetic relationship of *P. zhejiangensis* within Orchidaceae, a phylogenetic tree using the complete plastome sequences of 41 plant species were generated (Figure 3). The resulting ML tree had very high bootstrap support for the majority of clades, and the phylogenetic tree was shown to be consistent with the traditional morphology-based taxonomy of Orchidaceae. Nine plants from the genus *Phalaenopsis* formed a well-supported monophyletic clade with 100% bootstrap value, and *P. zhejiangensis* was sister to *P. wilsonii*, with a support value of 97%.

## Discussion

The classification of Orchidaceae is a very complex problem for its bewildering diversity and morphological parallelism, and also for botanical neglect (Dressler 1993). Orchidaceae were divided into five subfamilies, including Apostasioideae, Vanilloideae, Cypripedioideae, Orchidoideae and Epidendroideae, and Apostasioideae are monophyletic and sister to other subfamilies (Dixon et al. 2003; Tsai et al. 2013). The subfamily Orchidoideae is comprised of a large group of orchids, including four tribes (Diurideae, Cranichideae, Codonorchideae, and Orchideae) with around 3600 plant species that largely share terrestrial habits (Bateman et al. 2003; Serna-Sánchez et al. 2021). The genus *Phalaenopsis* belongs to tribe Orchideae, which consists of about 62 genera (Inda et al. 2012). The morphology of *P. zhejiangensis* is similar to both *Doritis* and *P. lowii*, with the characteristic feature of pollinia similar to that of *Doritis*, and its shapes of petals and columns similar to *P. lowii* (Tsi 1999; Christenson 2001). *P. zhejiangensis* was initially named *N. zhejiangensis* based on morphology, and then renamed as *D*. *zhejiangensis* (Yukawa and Kita 2005; Schuiteman 2012). Molecular analyses using ITS, *trnL* intron as well as *trnL-F* spacer prove that *N. zhejiangensis* is species nested in genus *Phalaenopsis* (Deng et al. 2015). Our molecular phylogenetic analyses confirm its systematic position, which indicates that *P. zhejiangensis* is sister to *P. wilsonii*, with a high support value.

The sizes of previously published *Phalaenopsis* cp genomes ranged from 144,607 bp (*P. lobbii*) to 148,964 bp (*P. phalaenopsis aphrodite* subsp. *formosana*) (Chang et al. 2006; Kim et al. 2016; Wang et al. 2019; Chen et al. 2020; Zhang et al. 2020). In our present study, the complete plastid genome of *P. zhejiangensis* is only 143,574 bp in length, which turns out to be the smallest among all known cp genome sequences in genus *Phalaenopsis*. The cp genomes of most flowering plants range from 120 kb to 160 kb, and the difference in genome size is mainly caused by contraction and expansion of the IR region (Goulding et al. 1996; Ingvarsson et al. 2003; Yao et al. 2020). The IR sizes of published *Phalaenopsis* plastid genomes are 24,719 (*P. lobbii*) (Wang et al. 2019) to 25,846 bp (*P. equestris*) (Jheng et al. 2012). In our present study, the length of *P. zhejiangensis* IR sequence is 24,464 bp, which is smaller than that of *P. lobbii*.

Plastid gene loss and pseudogenization are common phenomena in parasitic, semi-parasitic, and saprophytic plants (Molina et al. 2014; Li and Zheng 2018; Nie et al. 2019). In semi-parasitic plants of *Macrosolen cochinchinensis*, *M. tricolor*, and *M. bibracteolatus*, the *infA* and all the *ndh* genes were lost among the three species, and two genes, *ycf1* and *rpl2*, were found to be pseudogenes (Nie et al. 2019). *Gastrodia elata* is a saprophytic plant with extremely small cp genome in which many genes related to photosynthesis are missing, and it contains only 28 genes, including 20 protein-coding genes, three rRNAs, and five tRNAs (Park et al. 2020). However, cp gene loss and pseudogenization are rarer in photosynthetic species, because the plastid gene cannot simply be discarded (Magee et al. 2010; Wicke et al. 2011; Daniell et al. 2016). Interestingly, pseudogenization, truncation, and deletion of *ndh* genes in cp genomes are common in some cp genomes of photosynthetic orchid plants (Kim et al. 2015). Orchids like *Apostasia odorata*, *Sobralia callosa*, *Paphiopedilum armeniacum*, and *Phragmipedium longifolium* retain the complete set of *ndh* genes, whereas most of the *ndh* genes in *P. equestris*, *Dendrobium officinale*, and *D. catenatum* were found to be lost in their plastids (Kim et al. 2015; Lin et al. 2017). In this study, six *ndh* genes are pseudogenized, and another six *ndh* genes are missing from the plastid genome of *Phalaenopsis zhejiangensis*.

## Data Availability

The data that support the findings of this study are openly available in GenBank of NCBI at https://www.ncbi.nlm.nih.gov/nuccore/MZ326749. The associated BioProject, SRA, and Bio-Sample numbers are PRJNA734444, SRR14710950, and SAMN19491762, respectively.
